# Transcriptome Changes of *Mycobacterium marinum* in the Process of Resuscitation From Hypoxia-Induced Dormancy

**DOI:** 10.3389/fgene.2019.01359

**Published:** 2020-02-07

**Authors:** Jun Jiang, Chen Lin, Junli Zhang, Yuchen Wang, Lifang Shen, Kunpeng Yang, Wenxuan Xiao, Yao Li, Lu Zhang, Jun Liu

**Affiliations:** ^1^ State Key Laboratory of Genetic Engineering, Institute of Genetics, School of Life Science, Fudan University, Shanghai, China; ^2^ State Key Laboratory of Genetic Engineering, MOE Engineering Research Center of Gene Technology, School of Life Science, Fudan University, Shanghai, China; ^3^ Department of Molecular Genetics, University of Toronto, Toronto, ON, Canada

**Keywords:** transcriptional regulation, resuscitation, *M. marinum*, hypoxia, latency

## Abstract

Nearly one-third of the world's population is latently infected with *Mycobacterium tuberculosis* (*M. tb*), which represents a huge disease reservoir for reactivation and a major obstacle for effective control of tuberculosis. During latent infection, *M. tb* is thought to enter nonreplicative dormant states by virtue of its response to hypoxia and nutrient-deprived conditions. Knowledge of the genetic programs used to facilitate entry into and *exit* from the nonreplicative dormant states remains incomplete. In this study, we examined the transcriptional changes of *Mycobacterium marinum* (*M. marinum*), a pathogenic mycobacterial species closely related to *M. tb*, at different stages of resuscitation from hypoxia-induced dormancy. RNA-seq analyses were performed on *M. marinum* cultures recovered at multiple time points after resuscitation. Differentially expressed genes (DEGs) at each time period were identified and analyzed. Co-expression networks of transcription factors and DEGs in each period were constructed. In addition, we performed a weighted gene co-expression network analysis (WGCNA) on all genes and obtained 12 distinct gene modules. Collectively, these data provided valuable insight into the transcriptome changes of *M. marinum* upon resuscitation as well as gene module function of the bacteria during active metabolism and growth.

## Introduction

*Mycobacterium tuberculosis* (*M. tb*), the causative agent of tuberculosis (TB), is the leading cause of death due to an infectious disease globally, with an estimated 10 million new cases and 1.3 million deaths in 2017. There were an additional 300,000 deaths from TB among HIV-positive people. The success of *M. tb* as a leading pathogen is associated with its ability to infect and persist in the host. About 1.7 billion people, 23% of the world's population, are estimated to have a latent TB infection (LTBI), which is asymptomatic but can persist for decades (Stewart et al., 2003; North and Jung, 2004). About 5–10% of LTBI will eventually develop active disease, and host immunosuppression (e.g., HIV coinfection) markedly increases the risk of reactivation (Corbett et al., 2007). LTBI poses a major challenge to the effective control of TB because of the difficulty in treatment and the fact that LTBI represents a huge disease reservoir.

During LTBI, *M. tb* is thought to enter nonreplicative ‘dormant' states by virtue of its lowered or altered metabolism in response to hypoxia, nitrosative stress, and/or nutrient deprivation (Boshoff and Barry, 2005). Accordingly, much research has been focused on environmental conditions and genetic programs that induce bacteriostasis, and the most extensively studied culture condition is hypoxia (Wayne and Sohaskey, 2001; Rustad et al., 2009). It was shown that an immediate bacterial response (2 hr) was the coordinated upregulation of 47 *M. tb* genes under the control of the response regulator (DosR) and two sensor kinases (DosS and DosT), known as the DosR regulon (Sherman et al., 2001; Boon and Dick, 2002; Park et al., 2003). A second set of 230 genes, induced by longer hypoxia exposure (7 days), was also identified (Rustad et al., 2008). These genes, collectively known as the enduring hypoxic response (EHR), were DosR-independent genes (Rustad et al., 2008).

During the reactivation of LTBI, the dormant bacteria are believed to resuscitate and resume active growth and metabolism. A few recent studies have used reaeration of hypoxic cultures for *in vitro* modeling of reactivation or resuscitation (Veatch and Kaushal, 2018). Several regulatory proteins, such as transcription factor ClgR and sigma factors SigH and SigE, were found to play a role in *M. tb* resuscitation from hypoxia (Mcgillivray et al., 2015; Iona et al., 2016; Veatch et al., 2016).

Despite the progress, knowledge of the genetic programs used to facilitate entry into and *exit* from the nonreplicative dormant states remains incomplete. In this study, we examined the transcriptional changes of *Mycobacterium marinum* (*M. marinum*) at different stages of resuscitation from hypoxia-induced dormancy. *M. marinum* is a pathogenic *Mycobacterium* and the closest genetic relative of the *M. tb* complex. *M. marinum* is an excellent model through which to understand various aspects of host–pathogen interactions in *M. tb* pathogenesis. For example, *M. marinum* and *M. tb* share many virulence determinants, such as the ESX-1 secretion system (Tobin and Ramakrishnan, 2008) and lipid virulence factors phthiocerol dimycocerosates and phenolic glycolipids (Yu et al., 2012). As such, findings from the current study of *M. marinum* may be applicable to *M. tb*.

## Result

### RNA-Seq Analysis of *M. marinum* Recovered From Hypoxia

Larry Wayne and co-workers were the first to develop an *in vitro* model to mimic the hypoxic environment of the human granuloma (Wayne, 1977; Wayne and Hayes, 1996; Wayne and Sohaskey, 2001). In the Wayne model, a sealed, standing culture is incubated over an extended period while the bacteria deplete the available oxygen. The gradual depletion of oxygen leads to nonreplicating persistence states with a concomitant shift in gene expression and metabolism.

To gain insight into the genetic mechanisms that facilitate the exit of mycobacteria from the nonreplicative state, we grew *M. marinum* under hypoxia for 7 days using the Wayne model and then reaerated the cultures. At different time points thereafter (0, 0.5, 4, 12, 24, and 48 hr), *M. marinum* cultures were collected and subjected to RNA-seq analysis. The growth curve of the bacteria is shown in **Supplementary Figure 1**. A total of 18 samples were collected (three biological replicates at each time point) and analyzed.

**Figure 1:**
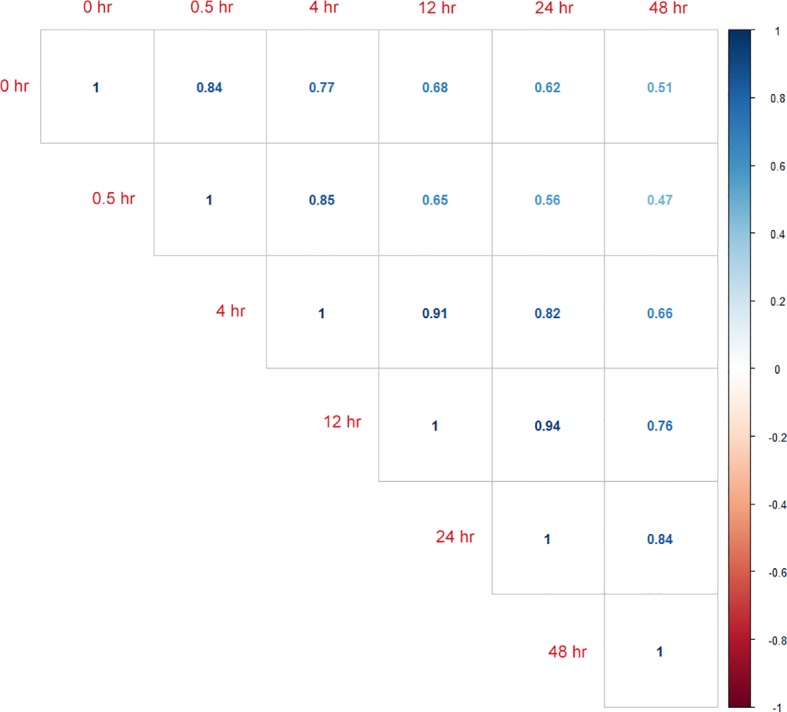
Calculated correlation coefficients between RNA-seq data from samples of different time points (0_hour, 0.5_hour, 4_hour, 12_hour, 24_hour, and 48_hour) during resuscitation.

The RNA-seq reads showed a high mapping ratio for all samples (>96%) (**Table 1**), supporting the overall sequencing accuracy. Transcripts of more than 4,900 genes were detected in each sample. We compared the RNA-seq data of cultures recovered at different time points under aerobic conditions. As expected, results showed that the correlation coefficient decreased as the interval between two samples increased (**Figure 1**). This result also suggested that the recovery from hypoxia is a gradual but dynamic process.

**Table 1:** RNA-seq reads of 18 samples (three biological replicates at each time point).

Sample	Total reads	Mapped reads	Pair mapped reads	Single mapped reads	Mapped ratio(%)
0hr_rep1	25667618	25206648	25071544	135104	98.2
0hr_rep2	24461374	24025951	23875630	150321	98.22
0hr_rep3	24356981	23991626	23851008	140618	98.5
0.5hr_rep1	27750348	27226045	27037288	188757	98.11
0.5hr_rep2	29227286	28693463	28508636	184827	98.17
0.5hr_rep3	30215819	29671934	29509385	162549	98.2
4hr_rep1	29552072	28996782	28795942	200840	98.12
4hr_rep2	25707932	25227133	25058798	168335	98.13
4hr_rep3	26459312	25956585	25781988	174597	98.1
12hr_rep1	20056376	19435701	19294860	140841	96.91
12hr_rep2	25849366	25305478	25143598	161880	97.9
12hr_rep3	24639687	24048335	23903266	145069	97.6
24hr_rep1	28399172	27857378	27662324	195054	98.09
24hr_rep2	31131638	30539305	30334952	204353	98.1
24hr_rep3	29686534	29152176	28950812	201364	98.2
48hr_rep1	28813928	28353514	28145540	207974	98.4
48hr_rep2	27427442	26995636	26807948	187688	98.43
48hr_rep3	28062635	27641695	27444061	197634	98.5

### Dynamic Changes of Gene Expression at Different Stage of Resuscitation

To analyze transcriptome changes of *M. marinum*, we focused on genes with RPKM ≥ 10 and compared samples from adjacent intervals: between 0.5 and 0 hr, 4 and 0.5 hr, 12 and 4 hr, 24 and 12 hr, as well as 48 and 24 hr. Differentially expressed genes (DEGs) were identified, and this was defined as a fold change greater than 2 and false discovery rate *P* value less than 0.05.

**Figure 2:**
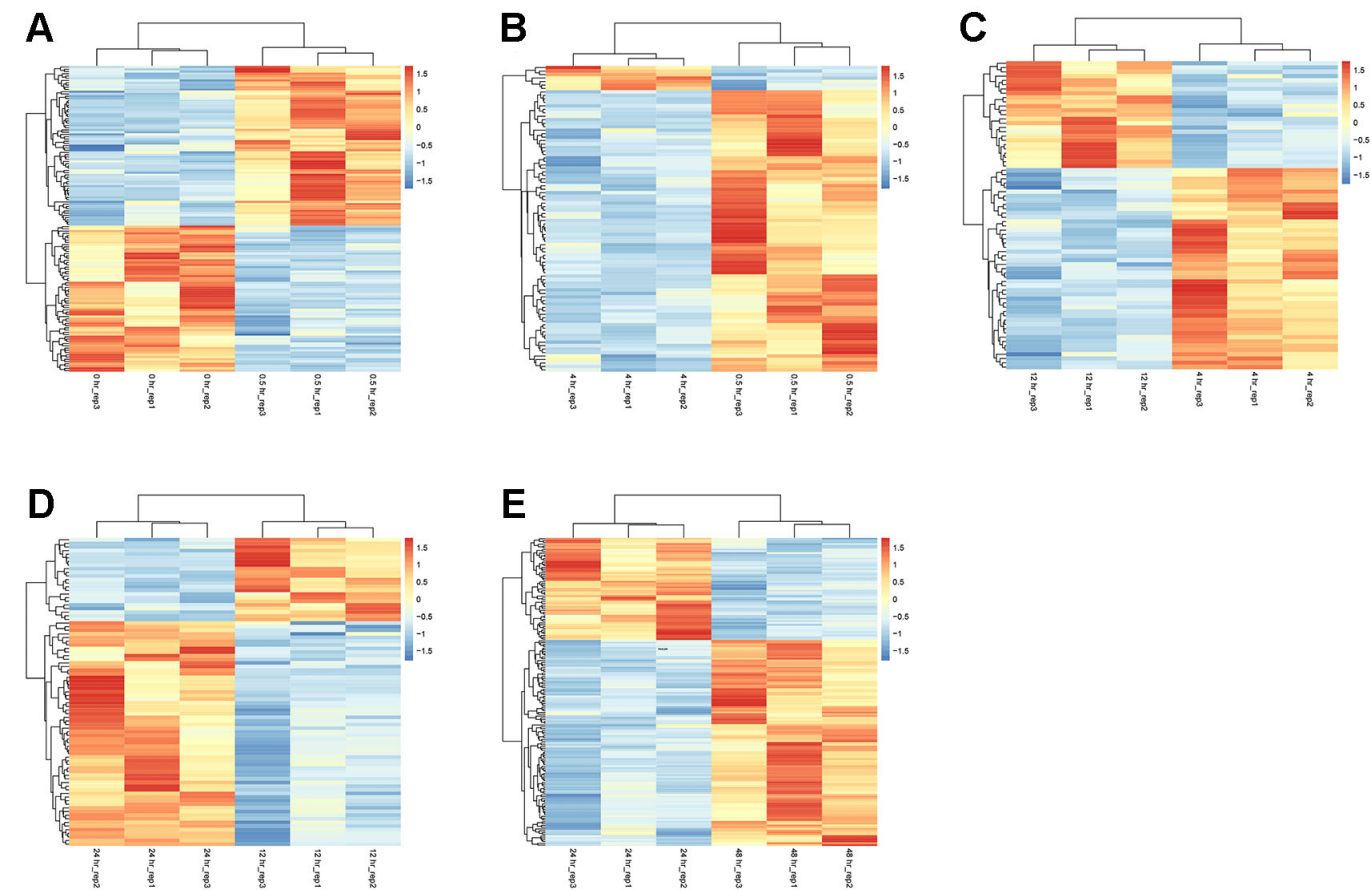
Heatmaps of DEGs between adjacent time points. At each time point, data from three biologically independent samples were included. **(A)** 0.5 vs. 0 hr; **(B)** 4 vs. 0.5 hr; **(C)** 12 vs. 4 hr; **(D)** 24 vs. 12 hr; and **(E)** 48 vs. 24 hr. The red color indicates upregulation. The blue color indicates downregulation.

At the earliest time point after resuscitation (0.5 hr), 136 DEGs were detected, of which 71 were upregulated and 65 were downregulated. Between 4 and 0.5 hr, most of the DGEs were downregulated (81 out of 88 total DEGs). The numbers of DGEs found between 12 and 4 hr, 24 and 12 hr, as well as 48 and 24 hr were 72, 85, and 172, respectively. The heat map of the five groups of DEGs is shown in **Figure 2**.

**Figure 3:**
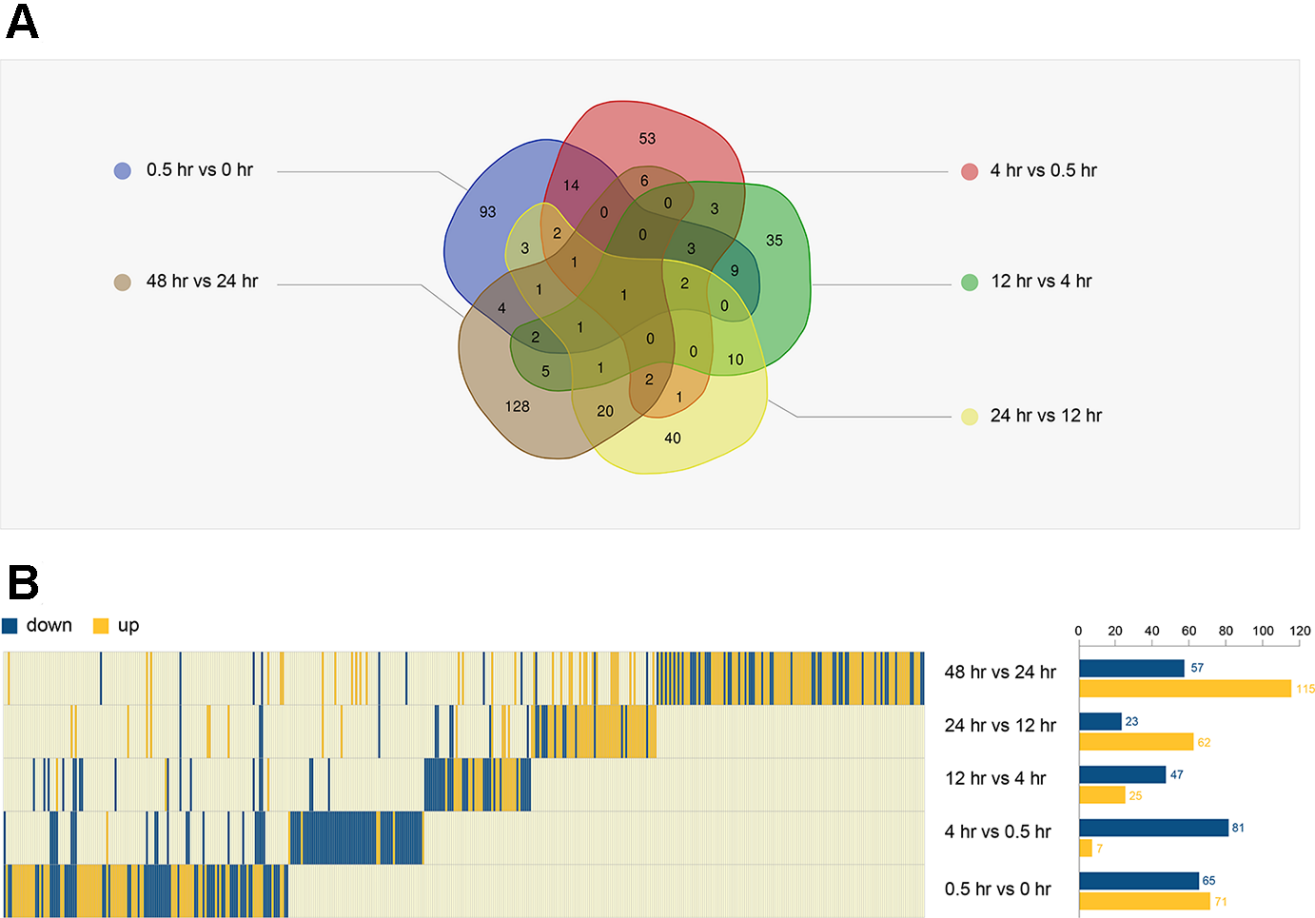
Analysis of DEGs in five groups. **(A)** The Venn diagram of five DEGs. **(B)** The expression trend of all DEGs (440 genes) in the five periods (left). The number of DEGs in each period is shown on the right.

We performed a Venn analysis of the five DEG groups (**Figure 3A**). The proportion of unique genes in each group was high: 68.4% (93/136, between 0.5 and 0 hr), 60.2% (53/88, between 4 and 0.5 hr), 46.7% (35/72, between 12 and 4 hr), 47.1% (40/85, between 24 and 12 hr), and 74.4% (128/172, between 48 and 24 hr). This suggests that a variety of genes were involved at different stages of resuscitation.

Combing the DEGs of different stages, a total of 440 genes were identified (**Supplementary Table 1**), and their expression underwent dynamic changes during resuscitation (**Figure 3B**). For example, the expression of *MMAR_0922*, *MMAR_3562*, and *MMAR_1654* were significantly changed at the early stage of resuscitation, suggesting that they may play an important role in this period. Some genes had changed in multiple time periods. For example, the expression of *MMAR_3403* was changed in last three periods, suggesting that this gene may be associated with the late stage of resuscitation.

### Validation of RNA-seq Results by RT-qPCR

**Figure 4:**
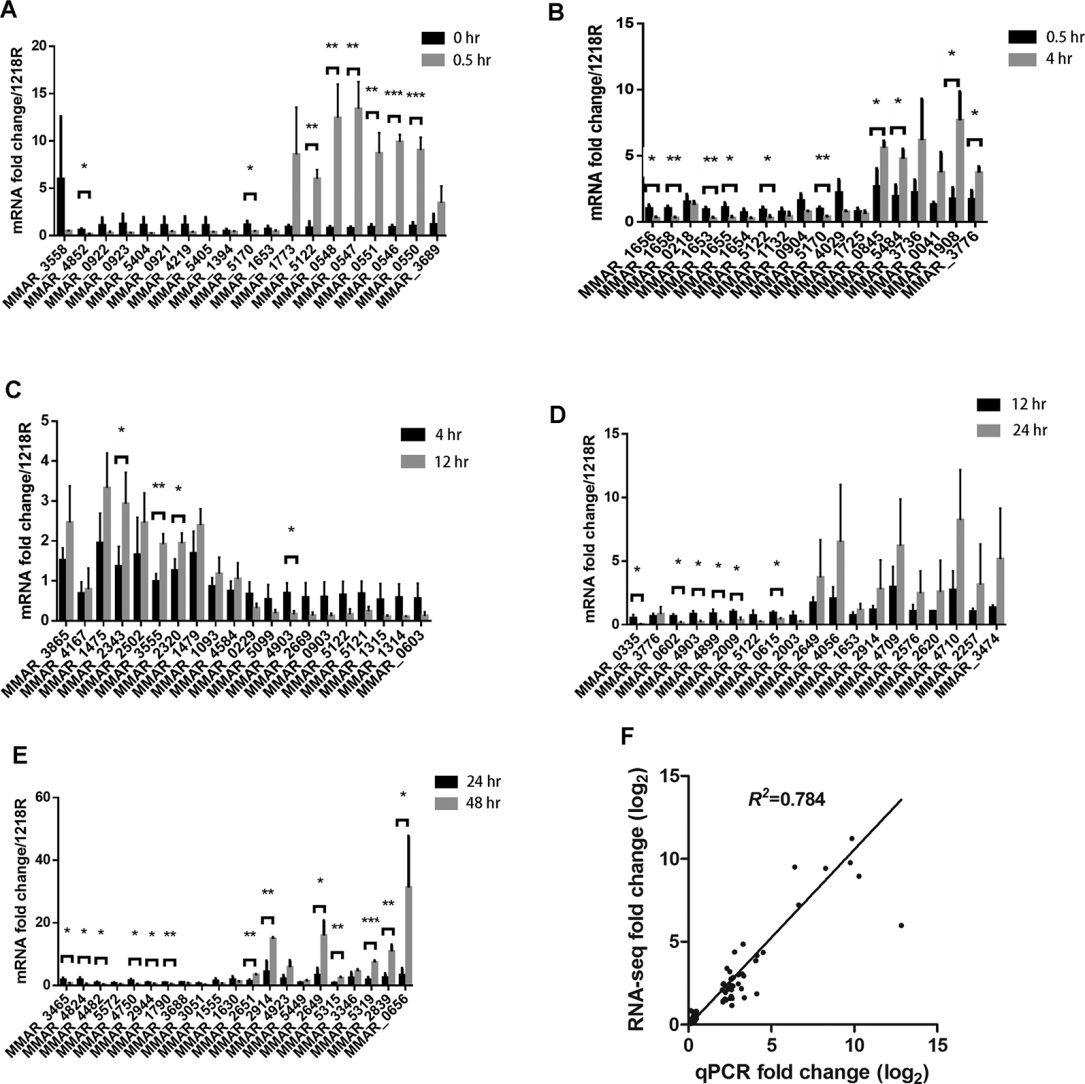
qRT-PCR results. **(A–E)** Approximately 20 genes from each period were selected and analyzed by qRT-PCR. *p < 0.05; **p < 0.01; and ***p < 0.001. **(F)** Scatter plot of RT-PCR data of all genes analyzed in **(A–E)**, comparing them to RNA-seq data of the same genes.

To validate the RNA-seq results, a real-time quantitative (RT-qPCR) analysis was performed. Three biologically independent samples at each time point were used for this experiment. For each DEG group, we selected the top 10 upregulated genes and 10 downregulated genes for this analysis (**Figure 4**). Between 0 and 0.5 hr, *MMAR_4852* and *MMAR_5170* (*whiB4*) were significantly downregulated, and six genes, *MMAR_5122* (*lipX*), *MMAR_0548* (*espG3*), *MMAR_0547* (*esxR*), *MMAR_0551* (*eccE3*), *MMAR_0546* (*esxG*), and *MMAR_0550* (*mycP3*), were significantly upregulated (**Figure 4A**). Between 0.5 to 4 hr, six genes, including *MMAR_1656*, *MMAR_1658* (*hycQ*), *MMAR_1653* (*Rv0081*), *MMAR_1655*, *MMAR_5122* (*lipX*), and *MMAR_5170* (*whiB4*), were significantly downregulated and four genes *MMAR_0845* (*hemB*), *MMAR_5484*, *MMAR_1908* (*ATC1*), and *MMAR_3776* (*rpfE*) were significantly upregulated (**Figure 4B**). Between 4 and 12 hr, *MMAR_2343* (*papA1*), *MMAR_3555,* and *MMAR_2320* (*wecE*) were significantly upregulated and *MMAR_4903* were significantly downregulated (**Figure 4C**). Between 12 and 24 hr, six genes, *MMAR_0335, MMAR_0602, MMAR_4903, MMAR_4899, MMAR_2009*, and *MMAR_0615 (iniA)*, were significantly downregulated (**Figure 4D**). Between 24 and 48 hr, six genes, *MMAR_3465* (*PPE51*)*, MMAR_4824, MMAR_4482* (*cypM*)*, MMAR_4750, MMAR_2944,* and *MMAR_1790* (*PPE2*), were significantly downregulated, and seven genes, *MMAR_2651, MMAR_2914* (*katG*)*, MMAR_2649, MMAR_5315* (*lpqH*)*, MMAR_5319, MMAR_2839* (*mpt63*), and *MMAR_0656*, were significantly upregulated (**Figure 4E**).

There is a good agreement between the RNA-seq and qPCR data, evident from the scatter plot using the expression levels of all 97 genes that were analyzed by both RNA-seq and qPCR (*R^2^^=^* 0.784) (**Figure 4F**). Based on this result, we consider that the RNA-seq data is reliable.

### Gene Ontology (GO) and Kyoto Encyclopedia of Genes and Genomes (KEGG) Pathway Enrichment Analysis

**Figure 5:**
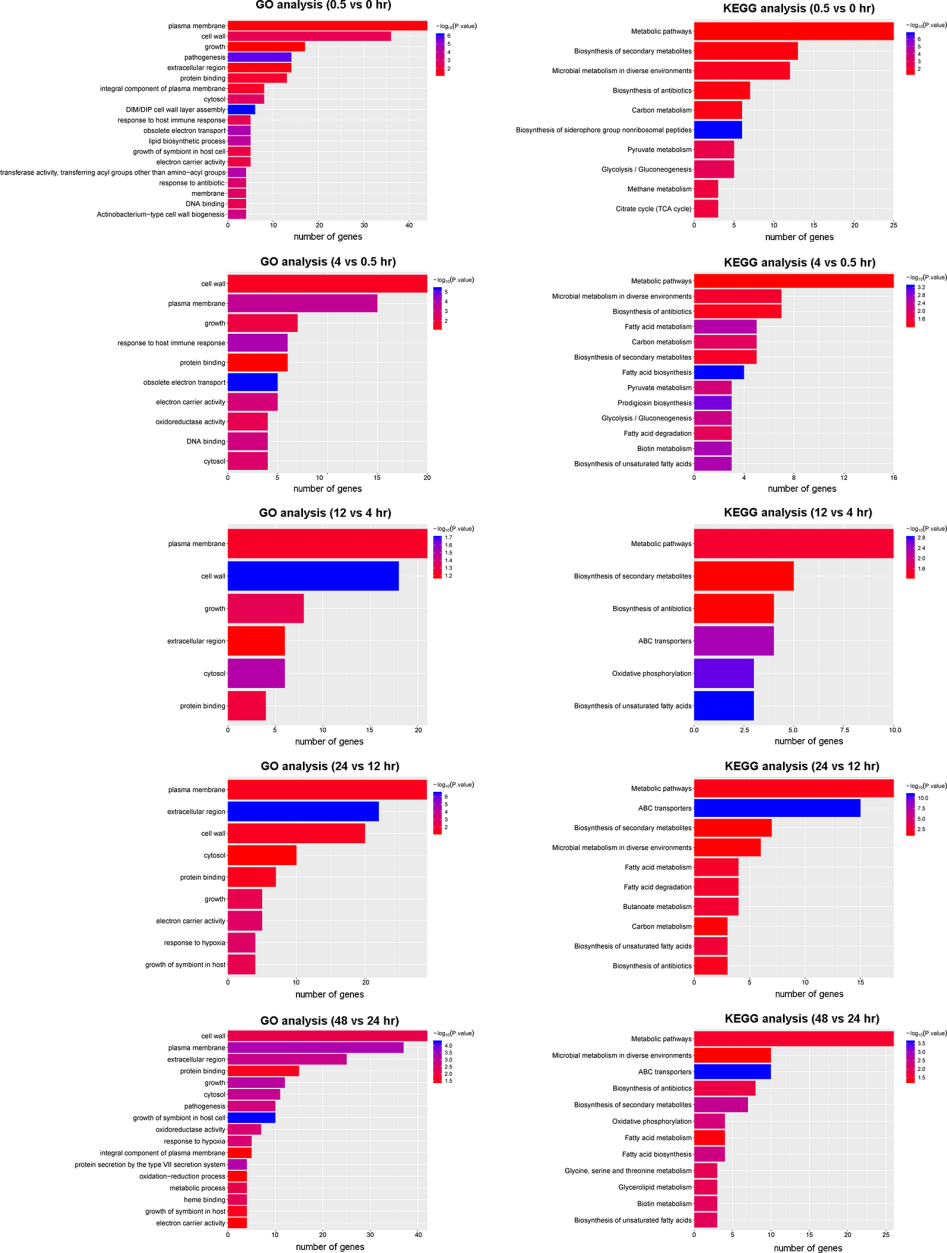
GO and KEGG analysis of DEGs in the five periods.

To gain insight into the biological consequence of the observed transcriptome changes, we performed GO and KEGG pathway analyses of the five DEG groups (**Figure 5**). A GO analysis was applied to identify the functional categories of DEGs. Between 0.5 and 0 hr, more than half of DEGs were involved in membrane and cell wall processes and were significantly enriched (**Figure 5**). This is consistent with the notion that, upon reaeration, the bacteria resumed cell division, which involved cell wall and membrane biogenesis. A KEGG analysis consistently revealed that DEGs involved in metabolic pathways and biosynthesis were significantly enriched and accounted for the largest number. A similar trend was observed for later periods, in which genes involved in cell wall and membrane biogenesis were highly enriched and accounted for the majority of DEGs at these stages. These results provide snapshots into the recovery of the bacteria from hypoxia and active growth under aerobic conditions.

### Co-Expression Analysis Between Transcriptional Regulators and mRNAs

**Figure 6:**
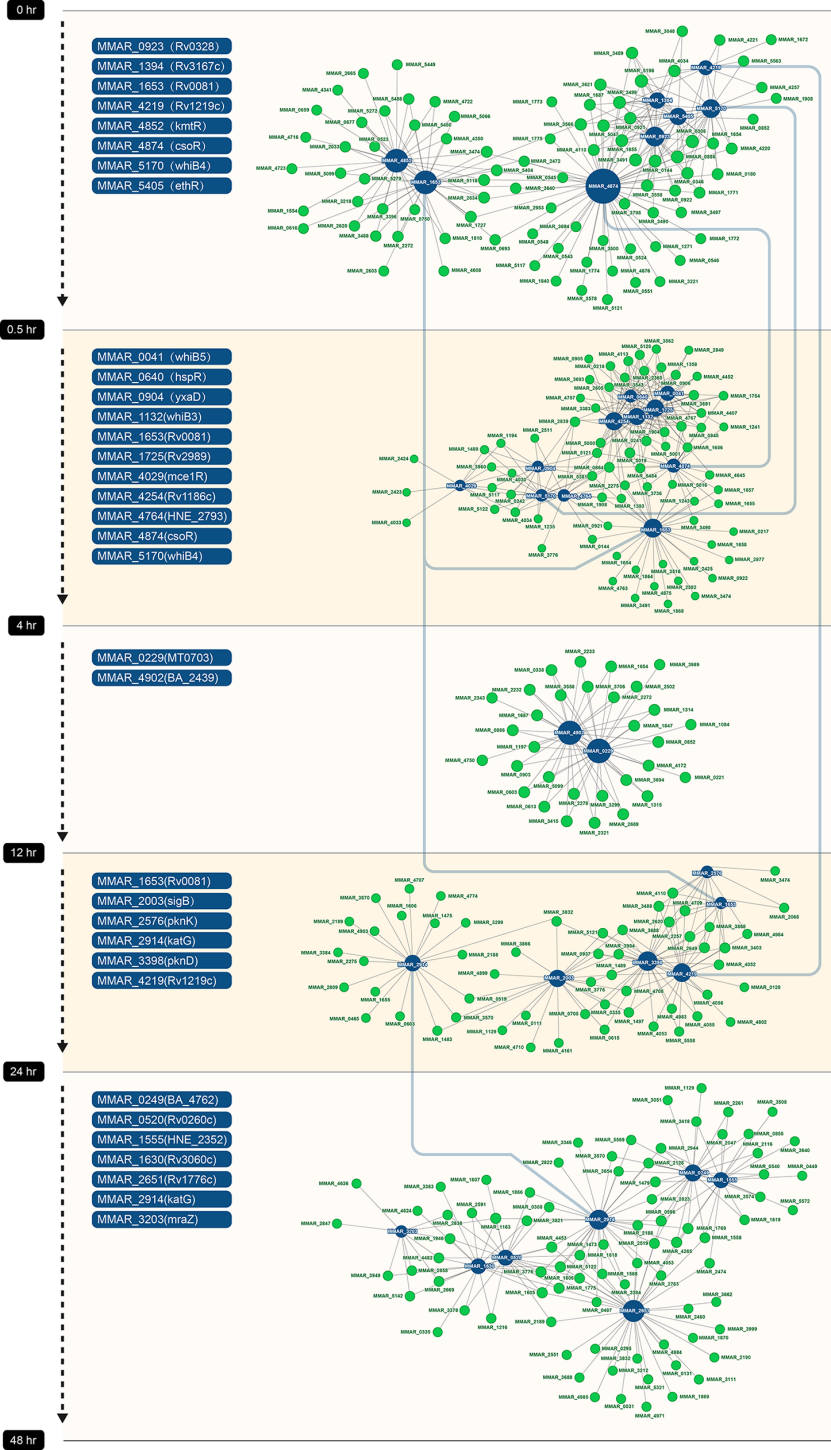
Co-expression networks in five periods. Each dot represents a gene. Transcription factors are labeled in blue, and other genes are labeled in green. Line between dots represents co-expression relationship between genes. The size of dot is proportional to the level of connectivity.

Co-expression networks can show relationships between genes. To explore the regulatory mechanisms at different stages of resuscitation, we constructed a co-expression network between transcriptional regulators and mRNAs. For this, we selected known transcriptional regulators, including transcription factors and sigma factors from the five DEGs, and calculated the correlation coefficients between these transcription factors and the remaining DEGs in the same group. We considered that a relationship existed between a given transcriptional regulator and other genes if the absolute value of the correlation coefficient was greater than 0.9, which included both positive and negative correlation. Based on these results, we constructed five co-expression modules and integrated them into a large network (**Figure 6**). The dark blue nodes in the figure represent transcription factors, and the green nodes denote DEGs in the same period. The size of the node is determined by the degree of connectivity. Greater degrees of connectivity are indicated by larger points. If there is a line between two nodes, then there is a relationship between them.

In the first co-expression module (between 0 and 0.5 hr), three transcription factors, MMAR_4874 (CosR), MMAR_1653 (Rv0081), and MMAR_4852 (KmtR), formed the major regulatory hubs, and MMAR_4874 (CosR) was the largest hub and interacted with other hubs in the network (**Figure 6**). The MMAR_4874 (CosR) and MMAR_1653 (Rv0081) hubs remained in the second co-expression module (between 0.5 and 4 hr), in addition to three new hubs formed by MMAR_4254, MMAR_1725 and MMAR_1132. In the third period (between 4 and 12 hr), MMAR_0229 and MMAR_4902 formed the hubs. In the fourth period (between 12 and 24 hr), MMAR_2003 (SigB) and MMAR_4219 formed the hubs. In the final period (24 to 48 hr), MMAR_2651, MMAR_1555, and MMAR_0249 formed the hubs.

### Weighted Gene Co-Expression Network Analysis (WGCNA)

The DEG analysis focused on partial dynamic changes during resuscitation. While the co-expression network of transcription factors provides an overview of the regulatory programs enabling the resuscitation of *M. marinum*, our knowledge on the overall genetic changes is still missing. Therefore, in this section, we analyzed the expression of all genes from cultures at different stages of resuscitation.

Weighted gene co-expression network analysis (WGCNA) (Langfelder and Horvath, 2008) is a method for analyzing the gene expression patterns of multiple samples. It clusters genes into modules by similar expression trends and reveals the relationship between gene modules and specific traits or phenotypes. We applied this method to analyze the RNA-seq data of *M. marinum* at different stages of resuscitation.

**Figure 7:**
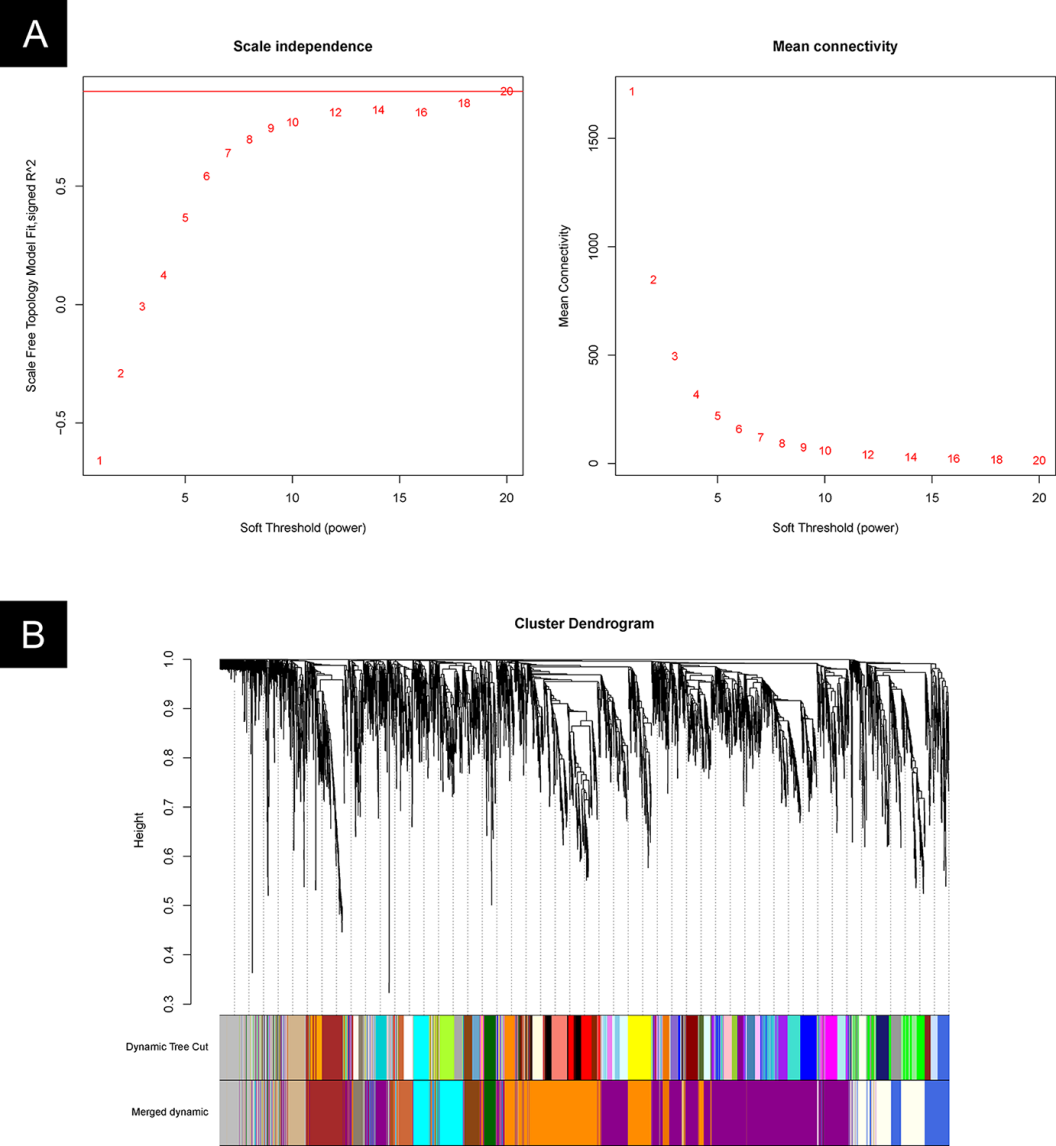
WGCNA cluster analysis. **(A)** Plot of the strength of correlation against a series (range 2 to 20) of soft threshold powers. **(B)** Gene clusters and gene module fusion.

The gene modules were identified by the WGCNA package in R software. We first determined the appropriate “soft-thresholding” value, which emphasizes strong gene–gene correlations at the expense of weak correlations. An optimal parameter (power = 20) was determined by plotting the strength of correlation against a series (range 2 to 20) of soft threshold powers (**Figure 7A**).

An unsigned pairwise correlation matrix was calculated, and the WGCNA algorithm was used to transfer the correlation coefficient between genes into the adjacent coefficient. Then, the dissimilarity of the topological overlap matrix was calculated based on the adjacent coefficient. Using the calculated dissimilarity, we carried out a hierarchical analysis by using agglomerative hierarchical clustering, also known as the bottom-up method. Other assumptions were made: (i) distances between different classes were measured by the average connectivity, and (ii) there should be at least 30 genes in each gene module.

Based on these analyses, we initially obtained 48 gene modules. The hierarchical cluster tree was then treated using the dynamic tree cut algorithm in the WGCNA package. A total of 13 gene modules were obtained. The “gray” module was the default module, which included discarded genes that could not be clustered. Thus, we focused on the analysis in the remaining 12 gene modules. The process of fusion is shown in **Figure 7B**. The number of genes varied among these 12 modules, and the detailed information is listed in **Table 2** and **Supplementary Table 2**.

**Table 2:** Information of gene modules identified by WGCNA.

Module_ID	Number of genes
module_34	1711
module_26	919
module_43	424
module_14	320
module_20	298
module_3	268
module_35	142
module_29	136
module_12	127
module_22	89
module_47	89
module_45	46

**Figure 8:**
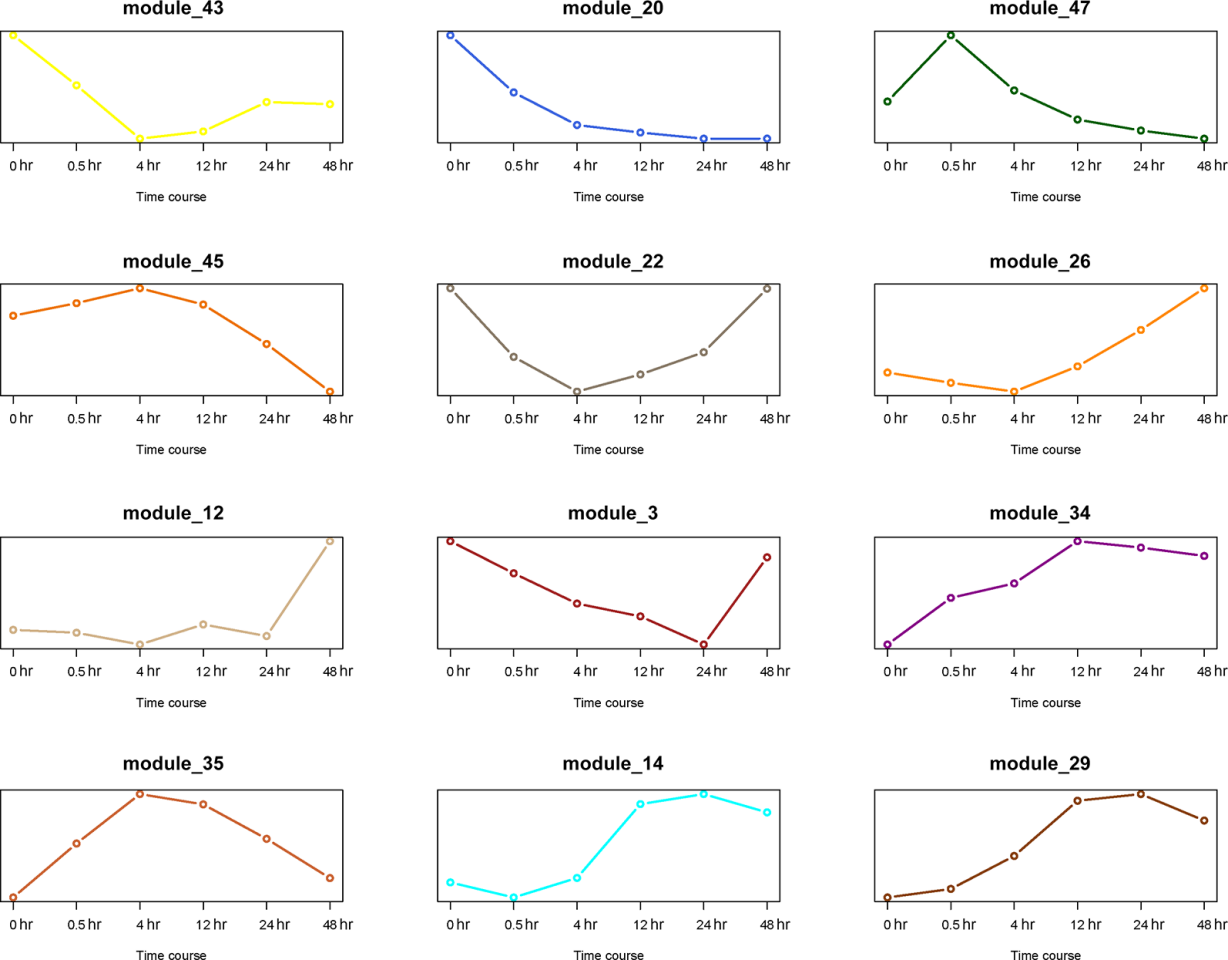
PCA analysis of 12 gene modules. The X axis represents time period, and the Y axis represents expression of first principal component.

The first principal component analysis (PCA) was performed on the 12 gene modules (**Figure 8**). The PCA results reflected the main trend of gene expression in the modules. Module 20 played an important role in the early stage (0 to 0.5 hr) of recovery, module 35 played a role mostly in the middle stage (4 to 12 hr), and module 12 was only involved in the last stage (24 to 48 hr). Other modules appeared to play roles in more extended periods.

### Identification of Key Gene Modules Associated With Different Stages of Resuscitation

**Figure 9:**
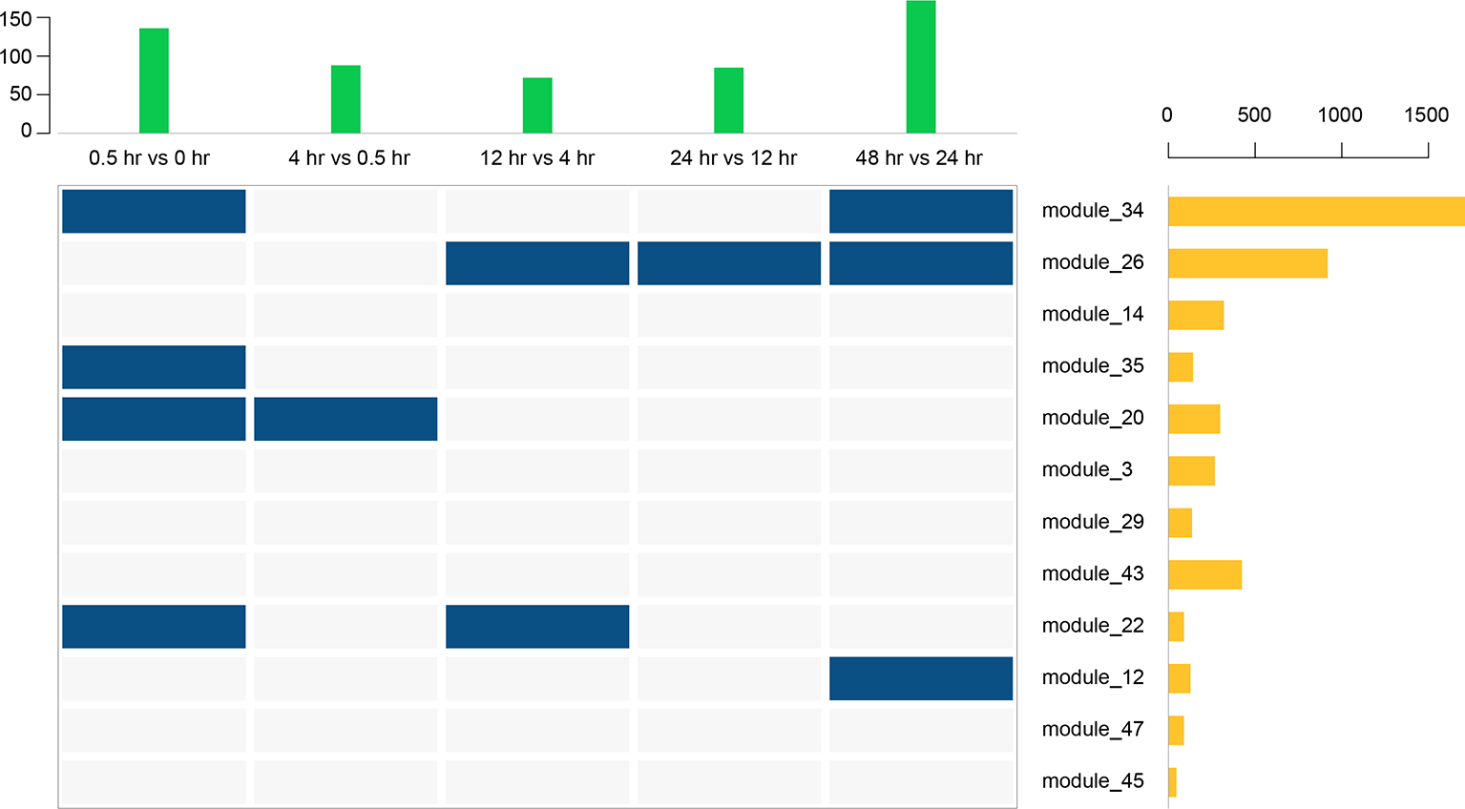
Enrichment analysis between 12 gene modules and 5 DEGs. The above bar chart represents the number of DEGs in each period, and the bar chart on the right represents the number of genes in each gene module. Blue squares represent a significant enrichment between the row set and the column set.

Thus far, we have identified 5 DEGs and 12 gene modules. We then performed an enrichment analysis between them. When the *P* value of Fisher's exact test was less than 0.001, we considered that these gene modules were significantly enriched in the DEG sets. The results are shown in **Figure 9**. Interestingly, module 20 was significantly enriched in DEGs of the early stage (0 to 0.5 hr) and module 12 was significantly enriched in DEGs of the last stage (24 to 48 hr) of the recovery. This is consistent with the result that these two modules were only involved in the early and last stages of resuscitation, respectively (**Figure 8**).

## Discussion

In this study, we examined the transcriptome changes of *M. marinum* recovered from hypoxia-induced dormancy. To gain a comprehensive view, multiple time points, including shortly after resuscitation (0.5 hr) to more extended periods up to 48 hr, were included. For each time point, three biologically independent samples were analyzed. Transcripts of the whole genome were analyzed by RNA-seq, and the quality of the RNA-seq data was reflected by the high genome mapping ratio and further validated by qPCR analysis of close to 100 genes. With these high-quality sequence data, we performed in-depth analyses, which included the identification of DEGs and the construction of co-expression network of transcription factors in each period. The availability of transcriptomes of independent samples at multiple time points also allowed us to employ a weighted gene co-expression network analysis to identify gene modules of *M. marinum*. Collectively, these data provide valuable insight into not only the genetic changes of the bacteria upon resuscitation but also the gene module function of *M. marinum* during active metabolism and growth.

A total of 136 DEGs were identified in *M. marinum* upon resuscitation from dormancy (0 to 0.5 hr), including eight transcription factors (**Figure 6**). Interestingly, all of these transcription factors were significantly downregulated. Among them, MMAR_1653 is a homolog of Rv0081 in *M. tb*. Previous studies have shown that Rv0081 is a member of the DosR regulon and is induced at the early stage of hypoxia (Sherman et al., 2001). Rv0081 is a major regulator of *M. tb* response to hypoxia and forms a large regulatory hub (Galagan et al., 2013; Chawla et al., 2018; Sun et al., 2018). Rv0081 plays an important role connecting the early and enduring hypoxic responses (Sun et al., 2018). WhiB4 (MMAR_5170) is an oxygen-sensitive transcription factor and has been shown to regulate PE/PPE family proteins, and it plays a role in *M. marinum* virulence (Chawla et al., 2012; Wu et al., 2017). CosR (MMAR_4874) is a copper-inducible transcriptional regulator, and the loss of *cosR* resulted in a hypoxia-type response with the induction of the DosR regulon (Talaat et al., 2004; Ward et al., 2008; Marcus et al., 2016). Given their roles in the hypoxic response, it is not surprising that Rv0081, WhiB4, and CosR underwent dynamic changes in expression upon resuscitation by reaeration. Two other transcription factors that were downregulated at this stage, MMAR_5405 (EthR) and MMAR_1394 (Rv3176c), belong to the TetR family transcription factors (Leiba et al., 2014; Sharma et al., 2017).

We also found that multiple members of the ESAT-6 family proteins were upregulated upon resuscitation (Harboe et al., 1996; Priscille et al., 2004), including EsxA (Sandra et al., 2010; Zhang et al., 2016), EsxB (Sandra et al., 2010), EsxG (Sweeney et al., 2011), EsxH (Alka et al., 2013; Portal-Celhay et al., 2016), EsxK, and EsxN (Zhigang et al., 2017). These proteins are components of the Type VII secretion systems, and many of them are important T cell antigens and play a critical role modulating the host–pathogen interactions (Abdallah et al., 2007).

From 0.5 to 4 hr after reaeration, 11 transcription factors were downregulated (**Figure 6**), including Rv0081, CsoR, and WhiB4 as mentioned above. Notably, the expression of two other WhiB family proteins (Averina et al., 2012), WhiB3 and WhiB5, were also significantly altered. WhiB3 responds to dormancy signals, including hypoxia and NO, and controls redox homeostasis of the bacteria (Priscille et al., 2004). WhiB5 responds to oxygen and controls the expression type VII secretion systems (Priscille et al., 2004). Consistently, we found that *whiB3* was downregulated while *whiB5* was upregulated at this stage of resuscitation.

During the period of 4 to 12 hr, we identified 72 DEGs, including 7 *PPE* family genes (*MMAR_5121, MMAR_1095, MMAR_1235, MMAR_1847, MMAR_1905, MMAR_2669,* and *MMAR_3989*). The PE/PPE family proteins play a critical role in mycobacterial pathogenesis (Fishbein et al., 2015). In addition, several genes of the Mce family were upregulated, including *MMAR_3865 (mce2B), MMAR_3868 (mce5E), MMAR_3867 (mce5D), MMAR_3866 (mce5C)*. The Mce family genes comprise four mammalian cell invasion factor (*mce*) operons (*mce1-4*), and some of these are involved in the invasion of host cells (Zhang and Xie, 2011).

A picture appears to have emerged from these analyses; in the early stage of resuscitation from the hypoxia-induced dormancy, transcription factors critical for a hypoxia-induced response are downregulated, and, as the recovery continues, genes important for virulence and host interactions are upregulated.

A WGCNA analysis revealed 12 distinct gene modules. Of particular interest is gene module 20, which was involved in the early stage of resuscitation only (**Figure 8**). This module comprises of ~300 genes, many of which have unknown functions or annotations. Future studies focusing on genes in this module may help to understand the molecular machinery enabling the exit of the bacteria from dormancy.

## Methods and Materials

### Bacterial Strain, Media, and Growth Conditions

*M. marinum* 1218R (ATCC 927) was grown in Middlebrook 7H9 broth to OD600~0.5, at which point they were aliquoted and cultured in screw-capped conical flasks at 30°C without additional oxygen. The hypoxic culture conditions were described previously by Wayne and Hayes (Wayne and Hayes, 1996). After 7 days in hypoxic conditions, the screw cap was replaced with a permeable membrane, and the rest of the conditions were unchanged. After aeration, samples were taken at 0 h, 0.5 h, 4 h, 12 h, 24 h, and 48 h, and an aliquot was used to measure the OD value (**Supplementary Figure 1**). The remaining samples were collected and snap frozen in liquid nitrogen for RNA sequencing.

### RNA Extraction, Illumina Sequencing, and RT-qPCR

*M. marinum* cultures were centrifuged at 4,500 × g for 5 min at room temperature and frozen on dry ice. The frozen cell pellets were resuspended in 1 mL TRIzol reagent (CW Bio). RNA extraction and illumina sequencing were performed as previously described (Wu et al., 2017). Raw data of RNA sequencing have been uploaded to the GEO database (BioProject ID : PRJNA588556).

For RT-qPCR validation of RNA-seq data, 1 µg RNA was reversed-transcribed to cDNA, which was then used as the template for RT-PCR analysis. The primers for analyzing the selected genes were listed in **Supplementary Table 3**.

### Transcriptome and Bioinformatics Analysis

The RNA-seq analysis and identification of differentially expressed genes (DEGs) were performed as previously described (Lee et al., 2019).

### GO and KEGG Analysis

We used the Kyoto Encyclopedia of Genes and Genomes (KEGG) database (Ogata et al., 2000) (www.genome.jp/kegg/) and the Gene Ontology (GO) database (Ashburner et al., 2000) (www.geneontology.org/) for our data analysis.

### Construction of the Gene Co-Expression Network

We calculated the correlation coefficient between identified transcription factors and other DEGs in each time period. The correlation coefficient value ranges from -1 to 1, representing a negative and positive correlation, respectively. We considered the expression of two genes as correlated if the absolute value of the correlation coefficient was larger than 0.8. The results were imported into Cytoscape 3.0 to generate the network map (Kohl et al., 2011).

### Weighted Gene Co-Expression Network Analysis (WGCNA)

We used the RNA-seq data from multiple time points (three biological independent samples at each time point) for WGCNA analysis. We used the WGCNA package to cluster gene modules as follows.

- Define gene co-expression similarity: calculate the similarity between any two genes using Pearson's correlation coefficient (Sij  =  |cor(i,j)|, the correlation coefficient of gene i and gene j), which then forms the correlation matrix (S  =  [S_ij_]).
- Define the exponential weighted value β: for any gene pair (i and j), apply the exponential adjacency function in the WGCNA algorithm to measure their relation index, namely, the exponential weighted β square of the correlation coefficient (a_ij_  =  power(S_ij_, β)  =  |S_ij_|^β^). Exponential weighted β is the power of the correlation coefficient. We selected β  =  5 after the analysis (fit value *R^2^* to approximately 0.9).
- Define a measure of node dissimilarity: after determining the adjacency function parameter β, the correlation matrix S  =  [S_ij_] is switched into the adjacency matrix A  =  [a_ij_] and converted into the topological overlap matrix Ω  =  [ω_ij_]. k_i_ or k_j_ indicate the sum of one node's adjacency coefficients. The node is a gene (i or j).
  $$\omega_{ij}=\frac{l_{ij}+a_{ij}}{\min\left\{k_{i},k_{j}\right\}+1-\;a_{ij}}$$
  $$\begin{array}{ccc} l_{ij}=\sum_{\mu }a_{i\mu }a_{\mu j} & k_{i}=\sum_{\mu }a_{i\mu } & k_{i}=\sum_{\mu }a_{i\mu } \end{array}$$
- Build hierarchical clustering tree to identify gene modules: the hierarchical clustering tree built using the dissimilarity coefficient $d_{ij}^{\omega }$ $(d_{ij}^{\omega }=1-\omega_{ij}$), and the different branches represent the gene modules.

### Enrichment Analysis and PCA Analysis

To determine whether one set of genes were more enriched in another set of genes, we used the Chi-square test or Fisher's exact test (Upton, 1992). First, the two sets of genes were taken and used to form a 2*2 contingency table. If there was a value less than or equal to five in the table, the Fisher's exact test was applied; otherwise, the Chi-square test was applied. When the p value was less than 0.05, the two sets were considered significantly enriched to each other. PCA analyses were performed by the princomp function in R software (version 3.5.1)

## Data Availability Statement

Raw data of RNA sequencing have been uploaded to the GEO database (BioProject ID:PRJNA588556).

## Author Contributions

JJ and CL performed the experiment. JJ, CL, and JL wrote the manuscript. YW and JZ prepared **Figures 1**–**9**. LS, KY, and WX prepared **Tables 1** and **2**. LZ, YL, and JL provided guidance for experiments. All authors reviewed the manuscript.

## Conflict of Interest

The authors declare that the research was conducted in the absence of any commercial or financial relationships that could be construed as a potential conflict of interest.

## Supplementary Material

Supplementary Figure 1 Growth curve of M. marinum after reaeration from hypoxic cultures.

Supplementary Table 1 Detailed information on the differentially expressed gene list at different time periods.

Supplementary Table 2 List of genes identified in 12 gene modules by WGCNA analysis.

Supplementary Table 3 Primer sequences of genes analyzed by RT-PCR.
