# Supplementary figures and images for: Transcriptome Changes of Mycobacterium marinum in the Process of Resuscitation From Hypoxia-Induced Dormancy
Source: Front Genet. 2020 Feb 7;10:1359. doi: 10.3389/fgene.2019.01359 (PMC7025489; doi:10.3389/fgene.2019.01359)

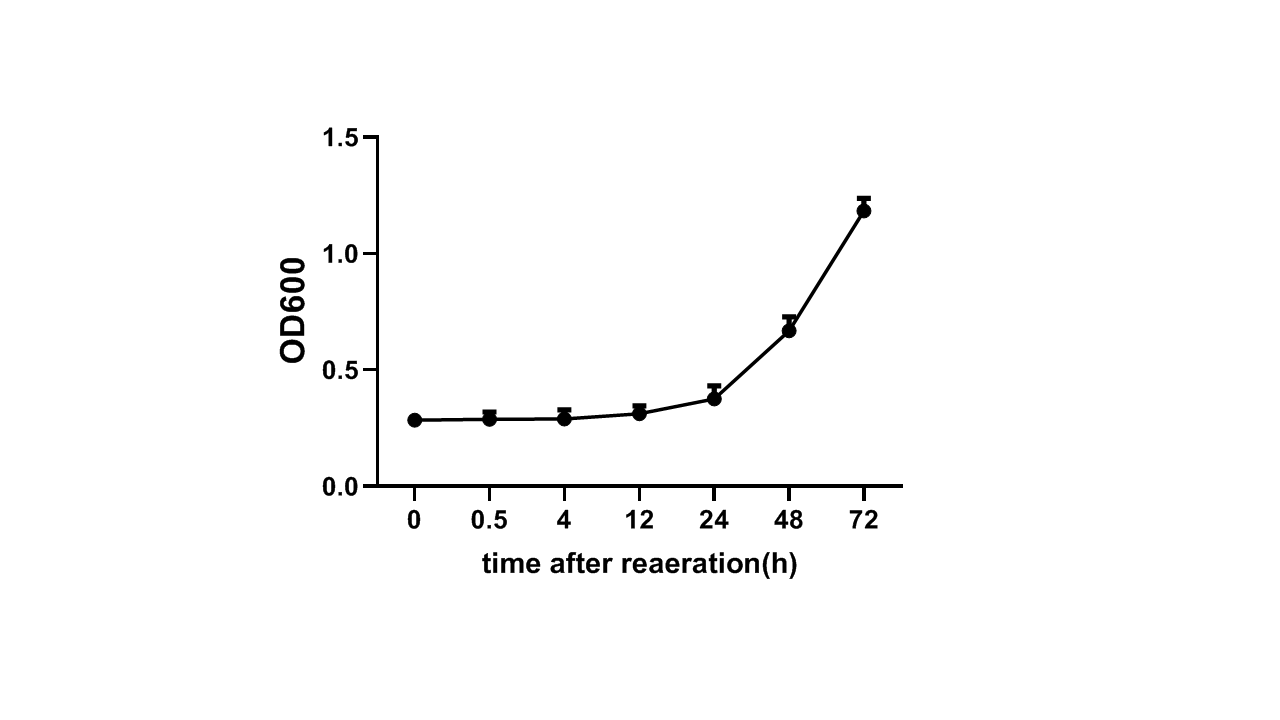

Supplement: Supplementary Figure 1 — Growth curve of M. marinum after reaeration from hypoxic cultures. [file Image_1.tif]
